# Economic burden of antibiotic resistance in China: a national level estimate for inpatients

**DOI:** 10.1186/s13756-020-00872-w

**Published:** 2021-01-06

**Authors:** Xuemei Zhen, Cecilia Stålsby Lundborg, Xueshan Sun, Nina Zhu, Shuyan Gu, Hengjin Dong

**Affiliations:** 1 Center for Health Policy Studies, School of Public Health, Zhejiang University School of Medicine, Hangzhou, China; 2 Centre for Health Management and Policy Research, School of Public Health, Cheeloo College of Medicine, (NHC Key Laboratory of Health Economics and Policy Research), Shandong University, Jinan, 250012 China; 3 Department of Global Public Health, Karolinska Institutet, Stockholm, Sweden; 4 National Institute for Health Research (NIHR) Health Protection Research Unit (HPRU) in Healthcare Associated Infections and Antimicrobial Resistance (HCAIs & AMR), Department of Infectious Disease, Imperial College, London, UK; 5 Center for Health Policy and Management Studies, Nanjing University, Nanjing, China; 6 The Fourth Affiliated Hospital Zhejiang University School of Medicine, Yiwu, China

**Keywords:** Antibiotic resistance, Multi-drug resistance, Economic burden, Inpatient, China

## Abstract

**Background:**

Antibiotic resistance (AR) threats public health in China. National-level estimation of economic burden of AR is lacking. We aimed to quantify the economic costs of AR in inpatients in China.

**Methods:**

We performed a multicentre and retrospective cohort study including 15,990 patient episodes at four tertiary hospitals in China from 2013 to 2015 to assess the impact of AR on hospital mortality, length of stay, and costs. We estimated the societal economic burden of AR using findings from the cohort study and secondary data from national surveillance hubs and statistical reports.

**Results:**

Patients with multi-drug resistant (MDR) infection or colonisation caused by *Staphylococcus aureus*, *Enterococcus faecalis*, *Enterococcus faecium*, *Escherichia coli*, *Klebsiella pneumonia*, *Pseudomonas aeruginosa*, and *Acinetobacter baumannii* experienced higher individual patient cost ($3391, 95% uncertainty interval (UI) $3188–3594), longer hospital stay (5.48 days, 95% UI 5.10–5.87 days), and higher in-hospital mortality rates (1.50%, 95% UI 1.29–1.70%). In China, 27.45% of bacterial infection or colonisation that occurred in inpatients were resistant, of which 15.77% were MDR. A societal economic burden attributed to AR was estimated to be $77 billion in 2017, which is equivalent to 0.37% of China’s yearly gross domestic product, with $57 billion associated with MDR.

**Conclusions:**

This is the first study to estimate national-level economic burden of AR in China. AR places a significant burden on patient health and healthcare systems. Estimation of economic costs of resistant infection or colonisation is the essential step towards building an economic case for global and national actions to combat AMR.

## Background

Antibiotic resistance (AR) threatens the effective prevention and treatment of an ever-increasing range of infections caused by bacteria and places one of the greatest threats on global health systems [1]. This problem is particularly severe in China, associated with overuse and misuse of antibiotics in human and animals [2, 3]. China was the second largest consumer of antibiotics in 2010 in the globe. In primary care facilities, 52.9% of outpatient prescriptions and 77.5% of inpatient prescriptions contained antibiotics, of which, only 39.4% and 24.6% were considered appropriate respectively [4]. Among BRICS countries (Brazil, Russia, India, China, and South Africa), more than 57.0% of the increase in antibiotic consumption in hospital occurred in China between 2000 and 2010 [5].

Driven by the inadequate consumption of antibiotics, China has the world’s most rapid growth of AR [6]. The proportion of methicillin-resistant *Staphylococcus aureus* (MRSA), third-generation cephalosporin resistant *Escherichia coli* (3GCREC), third generation cephalosporin resistant *Klebsiella pneumonia* (3GCRKP), carbapenem resistant *K. pneumonia* (CRKP), carbapenem resistant *Pseudomonas aeruginosa* (CRPA), carbapenem resistant *Acinetobacter baumannii* (CRAB) in China in 2017 were 32.2%, 54.2%, 33.0%, 9.0%, 20.7%, and 56.1%, respectively, which were higher than those in Europe (16.9%, 14.9%, 31.2%, 7.2%, 17.4%, and 33.4%) [7–9].

AR is associated with prolonged hospital stay, higher medical costs and increased hospital mortality [10]. In the European Union and European Economic Area countries, it was estimated that AR attributed to €1.1–1.5 billion economic loss 33,000 deaths each year [11, 12]. In the United States (US), AR led to approximately $55 billion excessive healthcare costs and subsequent societal costs and 35,000 deaths each year [7, 13].

However, in China, similar analysis to estimate the burden of AR on national level is lacking. Despite the recent recognition of the public health threat posed by AR and the development of a national action plan for antimicrobial resistance (AMR) [14], limited information as to its economic burden hinders the country’s progress in addressing AR [15]. Robust economic assessments of AR are urgently needed if the top-level political commitment is to be enforced [16]. In this study, we aimed to estimate the national level economic burden of AR in China for inpatients. First, we estimated the number of inpatients with AR in China; then, we estimated the societal economic burden for inpatients in China due to AR.

## Methods

### Study setting

We collected data in four tertiary hospitals in China, three in Zhejiang Province (Site 1, Site 3, and Site 4), and one in Shandong Province (Site 2). Site 1 and Site 2 are general provincial hospitals, Site 3 is a general county hospital, and Site 4 is a provincial hospital providing healthcare integrated with traditional Chinese medicine (Table 1). We selected these hospitals as study sites due to their relatively complete hospital information system, which can make reliable data collection possible.

**Table 1:** The characteristics of the study settings during 2013 and 2015

Characteristics	Site 1	Site 2	Site 3	Site 4
Province	Zhejiang	Shandong	Zhejiang	Zhejiang
Number of beds	3200	3500	1727	2100
Number of discharged inpatients yearly	170,000	160,000	80,000	50,000
Number of patients enrolled (% of inpatients with total positive bacterial infection or colonisation)	4541(60)	2535(40)	4993(100)	3921(100)
Number of patients with resistant infection or colonisation (SDR)	1112(20.37)	720(13.19)	1789(32.77)	1838(33.67)
Number of patients with resistant infection or colonisation (MDR)	2282(30.97)	1427(19.37)	1851(25.12)	1808(24.54)
Number of patients with susceptible infection or colonisation	1147(36.26)	388(12.27)	1353(42.78)	275(8.69)

*SDR* single-drug resistant, *MDR* multi-drug resistant

### Patient enrollment

This was a retrospective study. During the study period between January 2013 and December 2015, we extracted data from the electronic medical records (EMR) of the study sites for patients who had bacterial infection or colonisation confirmed by any clinical specimens (e.g. blood, urine, stool, cervical, or urethral sources) [17]. Only 60% of inpatients with positive samples from Site 1 and 40% cases from Site 2 were randomly selected due to the large inpatient population, and 100% cases from Site 3 and Site 4 were selected. We only included the first sample if there were multiple samples from the same isolate during the study period in order to avoid duplication. If a patient was re-admitted in the sampled hospitals, we recorded as multiple cases.

We extracted patient information, including patient demographics (age, sex, and health insurance), comorbidities (disease diagnosis, and Charlson Comorbidity Index (CCI)), hospital events (admitting service, surgical service, and date of hospital and intensive care unit (ICU) admission or discharge), and clinical outcomes (death or alive during the hospitalization when discharged) from first page of medical record system, microbiological data from microbiology laboratory, and costs for treatments from financial system. We matched different data from different system using patient identification, and patients with missing above information were excluded.

### Pathogen selection and case categorisation

In this study, we included patients with infection or colonisation caused by *S. aureus*, *E. faecalis*, *E. faecium*, *E. coli*, *K. pneumonia*, *P. aeruginosa*, and *A. baumannii*. We classified the infection or colonisation cases into susceptible episodes and resistant episodes (including resistant and intermediate isolates) based on the susceptibility test results for the patient specimens. The susceptible cases were in control group. The AR cases as case group were further categorised into either single-drug resistant (SDR) or multi-drug resistant (MDR). We defined SDR cases if patients were resistant to at least one antibiotic drug in one or two antibiotic categories, and MDR cases were defined as patients resistant to one antibiotic drug in three or more antibiotic categories. The interpretation of susceptibility test results was based on the Clinical and Laboratory Standards Institute (CLSI) definitions [8, 18].

### Statistical analysis

We conducted propensity score matching (PSM) to eliminate selection bias by balancing the potential confounding variables between susceptible and resistant infection or colonisation cases [19]. Patient demographics, comorbidities (cancer, diabetes or not), disease characteristics (CCI, number of diagnosis), and treatment (admission to ICU, surgery) were as independent variables. We employed 1:1 nearest-neighbor matching with a 0.05 caliper value, and the propensity score was balanced when there were no differences in baseline characteristics between the two groups. The outcome measure are individual patients’ hospital costs, length of hospital stay, and in-hospital mortality rate using matched pairs. We performed 1000 iterations of Monte Carlo simulations to calculate the 95% uncertainty interval (UI) for each outcome measure with normal distribution. Time discounting and age weighting were not considered in this study [20].

**Fig. 1:**
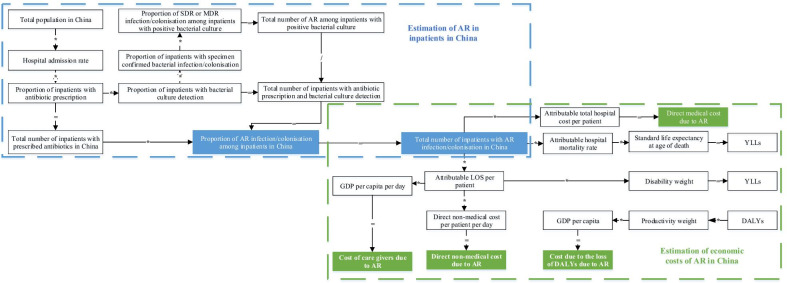
Flow chart to estimate national-level economic burden of antibiotic resistance in inpatients in China. *YLLs* years of life lost, *YLDs* years lived with disability, *DALYs* disability-adjusted life years, *GDP* gross domestic product, *SDR* single drug resistance, *MDR* multiple drug resistance, *AR* antibiotic resistance, *LOS* length of stay

The definition, value and data source of the input equation parameters are presented in Fig. 1. We parameterised the equation using data collected from the study sites before mentioned and data reported in published literature. We used the data from the latest year available for parameters from secondary data sources. If national level estimates are not available, we used the data collected from the study sites to represent the national population. The rates of MRSA, 3GCREC, FQREC, 3GCRKP, CRKP, CRPA, and CRAB among inpatients with positive bacterial culture from four sampled hospitals were 48.87%, 55.73%, 58.65%, 32.51%, 10.29%, 31.75%, and 56.54%, respectively, which are similar to the country’s average level reported by China Antimicrobial Resistance Surveillance System (34.42%, 57.17%, 52.82%, 35.05%, 8.03%, 22.62%, and 58.05%) [8]. The average total hospital cost, length of hospital stay, and in-hospital mortality rate in Zhejiang province and Shandong province in 2017 were $3252 and $2604, 9.8 days and 8.6 days, 0.3% and 0.4%, which were approximate to the national average ($2557, 9.3 day, and 0.4%) [21].

### Estimation of AR in inpatients in China

We estimated the number of inpatients with AR using primary data collected from the study cohort and secondary source data. We calculated total number of inpatients with AR infection or colonisation in China by multiplying total number of inpatients with prescribed antibiotics in China and proportion of AR infection or colonisation among inpatients in China. Except for total population in China and hospital admission rate, we did not consider age- and gender-specific attack rates due to sample size and data availability.

First, we extracted total population in China in 2017 [22] and hospital admission rate in 2013 [21] with age and gender distribution from United Nations (UN) and China Health Statistical Yearbook, respectively. Second, we collected proportion of inpatients with antibiotic prescription, proportion of inpatients with bacterial culture detection, and proportion of inpatients with specimen-confirmed bacterial infection or colonisation from National Nosocomial Infection Survey 2014. Third, proportion of SDR and MDR infection or colonisation among inpatients with positive bacterial culture was calculated using EMR from sampled hospitals between 2013 and 2015. Last, we calculated proportion of AR infection/colonisation among inpatients in China and total number of inpatients with AR infection/colonisation in China (Fig. 1, Additional file 1: Figure S1, Table S1, Table S2).

### Estimation of economic costs of AR in China

We calculated the economic costs of inpatients with AR by summing direct medical, direct non-medical, and indirect costs. All hospital costs were presented in 2015 US dollar values using the purchasing power parities and the 2015 consumer price index of China [23, 24]. Medical costs included out-of-pocket payment (by patients themselves) and payment covered by health insurers for medication and materials, diagnostic tests, treatment procedures, and other cost during a patient’s hospital stay. We calculated direct medical cost due to AR by multiplying attributable total hospital costs per patient and total number of inpatients with AR in China.

Attributable direct non-medical cost included costs for accommodation, meals, transport during the patient’s hospital stay. We used data from a previous survey for direct non-medical costs per day paid by individual hospitalised patients [25]. Then, we calculated direct non-medical cost due to AR by multiplying attributable direct non-medical stay per patient per day, length of hospital stay per patient and total number of inpatients with AR in China.

Attributable indirect costs included costs of care givers and productivity loss measured in disability-adjusted life years (DALYs) due to AR. Variables using to calculate DALYs included standard life expectancy at age of death from the UN [22], productivity weight and disability weight from Global Burden of Disease 2013, and GDP per capita in 2017 from China Statistical Yearbook [26]. Cost of care givers due to AR was calculated by multiplying GDP per capita per day, attributable length of hospital stay per patient and total number of inpatients with AR in China. Cost due to the loss of DALYs due to AR was calculated by multiplying DALYs, productivity weight and GDP per capita. Due to the small sample size, DALYs calculations used average mortality instead of age-specific mortality and DALYs is the sum of years of life lost (YLLs) (multiplying total number of inpatients with AR in China, attributable hospital mortality rate, and standard life expectancy at age of death) and years lived with disability (YLDs) (multiplying total number of inpatients with AR in China, attributable length of hospital stay per patient, and disability weight) (Fig. 1).

### Ethics approval

The institutional review board of Zhejiang University School of Public Health reviewed and approved this study. All patient data has been anonymised prior to analysis.

## Results

### Demographic characteristics of the study cohort

We collected 3163, 5459, and 7368 inpatients with susceptible, SDR and MDR infection or colonisation from four sampled hospitals, The demographic characteristics of the patients included in the study cohort are presented in Tables 2 and 3. Older age, higher proportion of insurance coverage, lower number of diagnostics, lower CCI, higher ICU admission rate, and higher surgical admission rate were associated with the risk of developing SDR-infection or colonisation. Older age, being female, higher proportion of insurance coverage, higher number of diagnostics, higher CCI, higher ICU admission rate were associated with the risk of developing MDR-infection or colonisation.

**Table 2:** Baseline characteristics of inpatients with SDR and susceptible infection or colonisation before and after PSM

Baseline characteristics	Before PSM			After PSM		
	Susceptible	SDR	P- value	Susceptible	SDR	P value
Number of inpatients, n	3163	5459		3135	3135	
Age in year, median (Min–Max)	68 (0–100)	73 (0–100)	< 0.000	68 (0–100)	69 (0–100)	0.994
Sex male, n (%)	1904 (60.2)	3324 (60.9)	0.525	1881 (60.0)	1873 (59.7)	0.837
Insurance, n (%)	2529 (80.0)	4727 (86.6)	< 0.000	2525 (80.5)	2526 (80.6)	0.975
Number of diagnosis, median (Min–Max)	6 (1–36)	6 (1–30)	0.0002	6 (1–36)	6 (1–30)	0.925
CCI, median (Min–Max)	5 (1–37)	5 (1–33)	< 0.000	5 (1–37)	5 (1–33)	0.815
Admission to ICU, n (%)	335 (10.6)	415 (7.6)	< 0.000	329 (10.5)	321 (10.2)	0.740
Surgery, n (%)	923 (29.2)	1161 (21.3)	< 0.000	904 (28.8)	920 (29.4)	0.656
Myocardial infraction, n (%)	92 (2.9)	125 (2.3)	0.077	90 (2.9)	85 (2.7)	0.701
Congestive heart failure, n (%)	620 (19.6)	959 (17.6)	0.019	603 (19.2)	611 (19.5)	0.798
Peripheral vascular disease, n (%)	45 (1.4)	48 (0.9)	0.019	41 (1.3)	39 (1.2)	0.822
Cerebrovascular diseases, n (%)	1428 (45.2)	2665 (48.8)	0.001	1423 (45.4)	1369 (43.7)	0.170
Dementia, n (%)	45 (1.4)	254 (4.7)	< 0.000	45 (1.4)	40 (1.3)	0.585
Chronic pulmonary disease, n (%)	969 (30.6)	1388 (25.4)	< 0.000	960 (30.6)	969 (30.9)	0.805
Connective tissue disease, n (%)	80 (2.5)	132 (2.4)	0.748	80 (2.6)	75 (2.4)	0.684
Mild liver disease, n (%)	109 (3.5)	194 (3.6)	0.794	109 (3.5)	122 (3.9)	0.383
Peptic ulcer disease, n (%)	69 (2.2)	141 (2.6)	0.244	69 (2.2)	65 (2.1)	0.727
Diabetes mellitus, n (%)	795 (25.1)	1546 (28.3)	0.001	795 (25.4)	813 (25.9)	0.603
Diabetes mellitus with chronic complication, n (%)	112 (3.5)	242 (4.4)	0.044	112 (3.6)	104 (3.3)	0.580
Moderate to severe chronic kidney disease, n (%)	212 (6.7)	447 (8.2)	0.012	211 (6.7)	217 (6.9)	0.764
Hemiplegia, n (%)	20 (0.6)	54 (1.0)	0.083	20 (0.6)	17 (0.5)	0.621
Solid tumor without metastases, n (%)	223 (7.1)	425 (7.8)	0.212	223 (7.1)	233 97.4)	0.627
Leukemia, n (%)	37 (1.2)	66 (1.2)	0.872	37 (1.2)	38 (1.2)	0.908
Malignant lymphoma, n (%)	24 (0.8)	50 (0.9)	0.446	24 (0.8)	27 (0.9)	0.673
Severe liver disease, n (%)	34 (1.1)	81 (1.5)	0.111	34 (1.1)	36 (1.2)	0.810
Metastatic tumor, n (%)	137 (4.3)	304 (5.6)	0.012	137 (4.4)	133 (4.2)	0.803

*SDR* single-drug resistant, *CCI* Chalson comorbidity index, *ICU* intensive care unit, *PSM* propensity score matching

**Table 3:** Baseline characteristics of inpatients with MDR and susceptible infection or colonisation before and after PSM

Baseline characteristics	Before PSM			After PSM		
	Susceptible	MDR	P-value	Susceptible	MDR	P-value
Number of inpatients, n	3163	7368		3048	3048	
Age in year, median (Min–Max)	68 (0–100)	72 (0–102)	< 0.000	68 (0–100)	70 (1–100)	0.955
Sex male, n (%)	1904 (60.2)	4168 (56.6)	0.001	1805 (59.2)	1815 (59.6)	0.794
Insurance, n (%)	2529 (80.0)	6122 (83.1)	< 0.000	2469 (81.0)	2424 (79.5)	0.148
Number of diagnosis, median (Min–Max)	6 (1–36)	6 (1–37)	< 0.000	6 (1–36)	6 (1–37)	0.945
CCI, median (Min–Max)	5 (1–37)	5 (1–39)	< 0.000	5 (1–37)	5 (1–28)	0.626
Admission to ICU, n (%)	335 (10.6)	1133 (15.4)	< 0.000	333 (10.9)	356 (11.7)	0.352
Surgery, n (%)	923 (29.2)	2239 (30.4)	0.215	899 (29.5)	965 (31.7)	0.067
Myocardial infraction, n (%)	92 (2.9)	197 (2.7)	0.499	87 (2.9)	95 (3.1)	0.547
Congestive heart failure, n (%)	620 (19.6)	1193 (16.2)	< 0.000	577 (18.9)	569 (18.7)	0.793
Peripheral vascular disease, n (%)	45 (1.4)	93 (1.3)	0.507	43 (1.4)	48 (1.6)	0.597
Cerebrovascular diseases, n (%)	1428 (45.2)	3444 (46.7)	0.132	1392 (45.7)	1410 (46.3)	0.644
Dementia, n (%)	45 (1.4)	277 (3.8)	< 0.000	45 (1.5)	45 (1.5)	1.000
Chronic pulmonary disease, n (%)	969 (30.6)	1451 (19.7)	< 0.000	867 (28.4)	894 (29.3)	0.445
Connective tissue disease, n (%)	80 (2.5)	189 (2.6)	0.915	80 (2.6)	96 (3.2)	0.221
Mild liver disease, n (%)	109 (3.5)	321 (4.4)	0.030	109 (3.6)	104 (3.4)	0.727
Peptic ulcer disease, n (%)	69 (2.2)	199 (2.7)	0.121	68 (2.2)	71 (2.3)	0.797
Diabetes mellitus, n (%)	795 (25.1)	2137 (29.0)	< 0.000	791 (26.0)	775 (25.4)	0.639
Diabetes mellitus with chronic complication, n (%)	112 (3.5)	293 (4.0)	0.286	112 (3.7)	108 (3.5)	0.784
Moderate to severe chronic kidney disease, n (%)	212 (6.7)	713 (9.68)	< 0.000	212 (7.0)	194 (6.4)	0.355
Hemiplegia, n (%)	20 (0.6)	101 (1.4)	0.001	20 (0.7)	17 (0.6)	0.621
Solid tumor without metastases, n (%)	223 (7.1)	729 (9.9)	< 0.000	223 (7.3)	216 (7.1)	0.729
Leukemia, n (%)	37 (1.2)	130 (1.8)	0.025	37 (1.2)	39 (1.3)	0.817
Malignant lymphoma, n (%)	24 (0.8)	85 (1.2)	0.066	24 (0.8)	22 (0.7)	0.767
Severe liver disease, n (%)	34 (1.1)	109 (1.5)	0.100	34 (1.1)	30 (1.0)	0.615
Metastasis tumor, n (%)	137 (4.3)	316 (4.3)	0.921	136 (4.5)	142 (4.7)	0.713

*MDR* multi-drug resistant, *CCI* Chalson comorbidity index, *ICU* intensive care unit, *PSM* propensity score matching

Differences in comorbidities were also observed between groups of SDR-, MDR- and susceptible infection or colonisation. We performed PSM for 3135 pairs of SDR- and susceptible infection or colonisation, and 3048 pairs of MDR- and susceptible infection or colonisation. After PSM, the characteristics mentioned above appeared to be insignificant (Tables 2, 3).

### Number of inpatients with AR in China

**Fig. 2:**
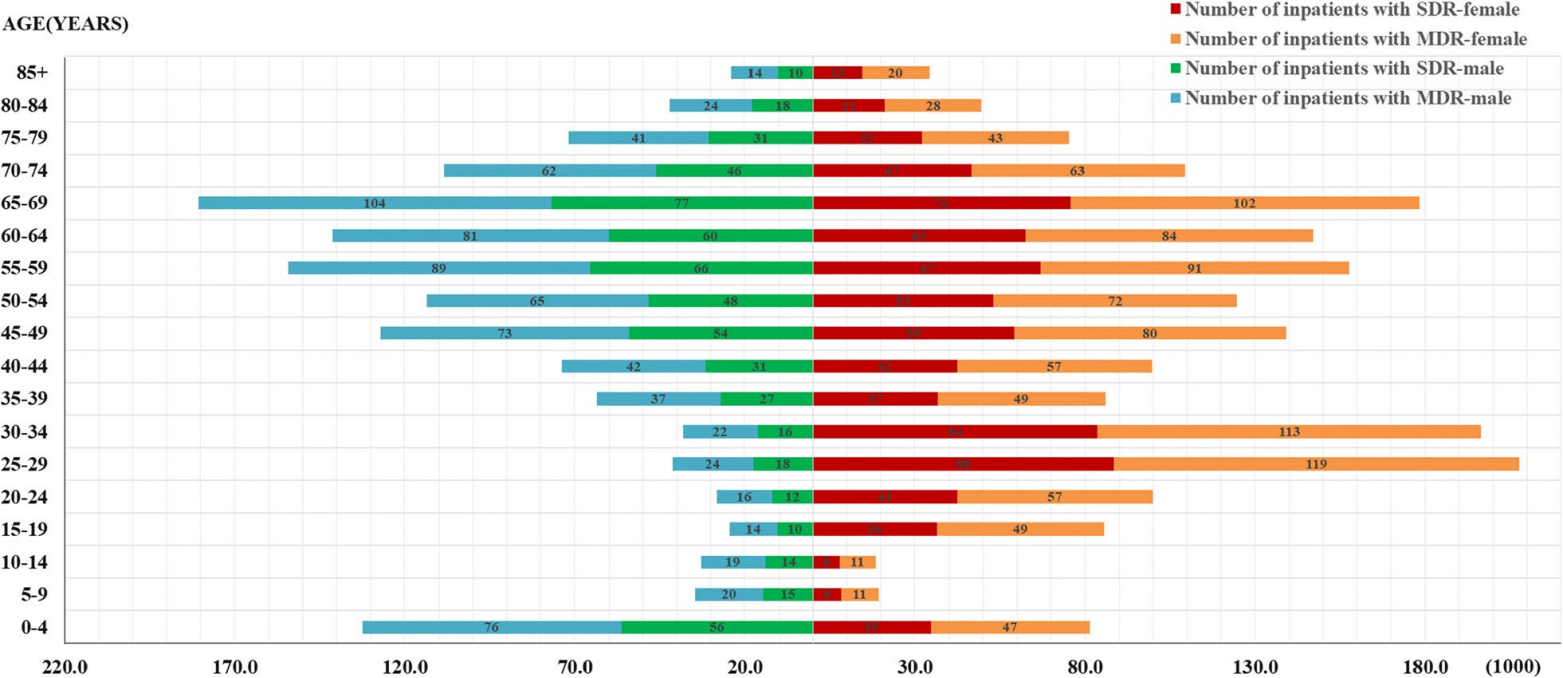
The estimated number of inpatients with antibiotic resistance in China. *SDR* single-drug resistance, *MDR* multiple-drug resistance

The secondary data used to estimate the national-level proportion of AR in China was presented in Additional file 1, including total population with gender and age distribution (Additional file 1: Figure S1), hospital admission rates aggregated by gender and age group (Additional file 1: Table S1), and proportion of bacterial infection or colonisation (Additional file 1: Table S2). We estimated 12,098,752 inpatients (27.45% of all inpatients) had AR infection or colonisation nationwide, including 5,113,276 (11.68%) with SDR and 6,985,476 (15.77%) with MDR among all among inpatients using the secondary data before mentioned and the proportion of susceptible, SDR and MDR infection and colonisation in our study cohort (Fig. 2).

### Economic costs associated with AR

Compared with patients with susceptible infection or colonisation, the mean differences in total hospital cost, length of hospital stay, and in-hospital mortality rate were $1144 (95% UI $965–$1322), 4.09 days (95% UI 3.70–4.47 days), 0.78% (95% UI 0.59–0.96%) in patients with SDR infection or colonisation, and $3391 (95% UI $3188–$3594), 5.48 days (95% UI 5.10–5.87 days), 1.50% (95% UI 1.29–1.70%) in patients with MDR infection or colonisation (Table 4, 5). In addition, GDP per capita in 2017 and direct non-medical cost per person was $15,011 [21] and $88 (95% UI $85–$91), respectively. We set productivity weight as 0.15, 0.75, 0.80, 0.1 for aged 0–14 years, 15–44 years, 45–59 years, above 60 years, respectively [27]. Disability weight was derived from Global Burden of Disease 2013 with a value of 0.133 (95% UI 0.332–0.190) [22]. Standard life expectancy at age of death was presented in Table S3 [22].

**Table 4:** Total hospital cost, length of hospital stay, and in-hospital mortality of inpatients with SDR and susceptible infection or colonisation

Inpatients	Total hospital cost ($)			Length of hospital stay (days)			In-hospital mortality rate (%)		
	Mean	95% UI		Mean	95% UI		Rate	95% UI	
Susceptible	9558	9432	9684	22.01	21.72	22.29	1.92	1.80	2.04
SDR	10,702	10,576	10,827	26.07	25.81	26.34	2.67	2.53	2.82
Difference	1144	965	1322	4.09	3.70	4.47	0.78	0.59	0.96

*SDR* single-drug resistant, *UI* uncertainty interval

**Table 5:** Total hospital cost, length of hospital stay, and in-hospital mortality of inpatients with MDR and susceptible infection or colonisation

Inpatients	Total hospital cost ($)			Length of hospital stay (days)			In-hospital mortality rate (%)		
	Mean	95% UI		Mean	95% UI		Rate	95% UI	
Susceptible	9616	9492	9739	22.20	21.91	22.48	2.08	1.95	2.20
MDR	13,017	12,857	13,176	27.70	27.44	27.96	3.58	3.41	3.74
Difference	3391	3188	3594	5.48	5.10	5.87	1.50	1.29	1.70

*MDR* multi-drug resistant, *UI* uncertainty interval

We estimated a total societal economic cost attributed to AR in inpatients in China of $77 billion (95% UI $67 billion–$87 billion), including $35 billion (95% UI $32 billion–$38 billion) of direct cost and $42 billion (95% UI $35 billion–$49 billion) of indirect cost. The attributable total economic cost is equivalent to 0.37% of China’s GDP in 2017, among which, $20 billion (95% UI $16 billion–$24 billion) was caused by SDR infection or colonisation, and $ 57 billion (95% UI $ 51 billion–$ 63 billion) by MDR infection or colonisation (Table 6).

**Table 6:** Economic burden caused by inpatients with SDR and MDR infection or colonisation in China

Economic burden ($ billion)	SDR			MDR			ABR		
	Mean	95% UI		Mean	95% UI		Mean	95% UI	
*Direct economic burden*									
Direct medical cost	6	5	7	24	22	25	30	27	32
Direct non-medical cost	2	2	2	3	3	4	5	5	6
Direct economic burden	8	7	9	27	25	29	35	32	38
*Indirect economic burden*									
Cost of productivity loss measured in DALYs	11	8	13	28	24	32	39	32	45
Cost of care giver	1	1	1	2	2	2	4	3	4
Indirect economic burden	12	9	15	30	26	34	42	35	49
*Societal economic burden*									
Socio-economic burden	20	16	24	57	51	63	77	67	87
Socio-economic burden accounted for GDP (%)	0.10	0.08	0.11	0.27	0.25	0.30	0.37	0.32	0.42

*SDR* single-drug resistant, *MDR* multi-drug resistant, *ABR* antibiotic resistant, *UI* uncertainty interval, *DALYs* disability-adjusted life years, *GDP* gross domestic product

## Discussion

Quantifying the economic costs of resistant infection or colonisation is the essential step towards building an economic case for global and national actions to combat AMR. To our knowledge, this study is the first study to estimate economic burden of AR in China at national level.

We estimated a percentage of 27.45% inpatients with resistant infection or colonisation, which is similar to the figure reported in previous studies conducted on regional level in China [6]. We predicted a total number of 7.0 million inpatients with MDR infection or colonisation in China, which is significantly higher than the number of 2.8 million in the US [13] and of 0.7 million in Europe [11]. In addition, The attributable economic burden associated with AR in China was $77 billion, which caused the similar GDP loss in China and the US despite the diversity in healthcare system settings, suggesting that AMR poses threat to all economies globally and cross-border cooperation is critical to mitigate the negative impact of AMR [10].

Since 2015, a series of national guidelines and recommendations for prudent use of antibiotics was launched to demonstrate the nation’s top-level political commitment, including China’s National Action Plan of AMR in humans (2016–2020), National Action Plan of AMR in animals (2017–2020), National Administrative Regulations for Clinical Use of Antibacterial Agents, and a national campaign in public hospitals for AR [28, 29]. Significant progress has been made in achieving some of the objectives in the National Action Plan of AMR. Legislative enforcement in antibiotic use in paediatric patients and food-producing animals, and national surveillance with timely mandatory reporting and data accessibility highlighted the country’s progress, reflected by the reduction in broad-spectrum antibiotic consumptions for surgical prophylaxis and among inpatients; and decrease in total hospital resistance and MRSA incidence [30]. Tailored training and research has been embedded in hospitals to guide clinical practice and minimise skill and knowledge gaps in healthcare professionals [30]. However, areas for improvement remain. AMR is absent from the nation’s wider public health agenda, including Healthy China 2030, which lacks any strategic measure to address AMR. Shortages of qualified general practitioners and low utilisation of nursing workforce hindered local implementation of top-level policy directive [31, 32]. Organisational regulation relies on administrative power rather than dedicated professional roles. Further, pharmaceutical industry continues incentivising prescription and over-the-counter sales [33]. Low public awareness, driven by limited health literacy about anti-infective and anti-inflammatory drugs, remains a barrier to engaging patients and citizens to promote optimal antimicrobial stewardship and infection prevention [22]. To overcome the challenge, it is essential to establish estimation of the burden of AMR on both public health and economic systems. Our study made contribution in both empirical knowledge and methodology for future research of AMR in China.

Our study has four limitations, which provide the scope for future studies. First, this study is focused on methodology to discuss how to scientifically estimate the economic burden of AR in China, the results may not be the accurate answer to the current economic burden of AR in China. Some variables, such as total hospital cost, length of hospital stay, and in-hospital mortality rate were collected from four tertiary hospitals in this study, and may have limited representativeness when used to predict national level economic burden. To minimise the bias, we validated the data in resistance level using historical statistics on national level. Second, the data is only valid for patients seeking care in the hospital, the impact of AR in the community has not been included. Third, due to the retrospective nature of our study, it is difficult to distinguish between colonisation and infection, which may lead to underestimation of the clinical and economic outcomes of AR infection. AR also impacts patients who do not become infected, and some studies reported that colonisation is associated with increased hospital cost, hospital stay, and hospital mortality [10], therefore, colonisation, as an important reservoir for bacteria causing infection, should be considered as well. Moreover, we only included observed variables in the PSM method, and some hidden bias may remain after matching.

## Conclusion

We estimated a percentage of 27.45% inpatients with resistant infection or colonisation nationwide in China, of which MDR accounted for 15.77%. we estimated a total societal economic cost attributed to AR in inpatients in China of $77 billion, which is equivalent to 0.37% of China’s GDP in 2017. AR places a significant burden on patient health and healthcare systems. Context-specific interventions are urgently needed to be implemented to promote prudent use of antibiotics in hospitals in China. Quantifying the economic costs of resistant infection or colonisation is the essential step towards building an economic case for global and national actions to combat AMR. It is essential to establish estimation of the burden of AMR on both public health and economic systems. Our study made contribution in both empirical knowledge and methodology for future research of AMR in China.

## Supplementary Information

**Additional file 1**.

## Data Availability

All data analysed during this study are provided in the Tables 1–6 and Figs. 1–2, and Additional file 1.

## Abbreviations

AR: Antibiotic resistance
MRSA: Methicillin-resistant *Staphylococcus aureus*
VREfm: Vancomycin-resistant *Enterococcus faecium*
VREfs: Vancomycin-resistant *Enterococcus faecalis*
3GCREC: Third-generation cephalosporin resistant *Escherichia coli*
3GCRKP: Third generation cephalosporin resistant *Klebsiella pneumonia*
CRKP: Carbapenem resistant *Klebsiella pneumonia*
CRPA: Carbapenem resistant *Pseudomonas aeruginosa*
CRAB: Carbapenem resistant *Acinetobacter baumannii*
GDP: Gross domestic product
US: United States
AMR: Antimicrobial resistance
EMR: Electronic medical records
CCI: Charlson Comorbidity Index
ICU: Intensive care unit
SDR: Single-drug resistant
MDR: Multi-drug resistant
PSM: Propensity score matching
UI: Uncertainty interval
UN: United Nations
DALYs: Disability-adjusted life years
