# Correction to: Economic burden of antibiotic resistance in China: a national level estimate for inpatients

**DOI:** 10.1186/s13756-021-00934-7

**Published:** 2021-04-01

**Authors:** Xuemei Zhen, Cecilia Stålsby Lundborg, Xueshan Sun, Nina Zhu, Shuyan Gu, Hengjin Dong

**Affiliations:** 1 Center for Health Policy Studies, School of Public Health, Zhejiang University School of Medicine, Hangzhou, China; 2 Centre for Health Management and Policy Research, School of Public Health, Cheeloo College of Medicine (NHC Key Laboratory of Health Economics and Policy Research), Shandong University, Jinan, 250012 China; 3 Department of Global Public Health, Karolinska Institutet, Stockholm, Sweden; 4 National Institute for Health Research (NIHR) Health Protection Research Unit (HPRU) in Healthcare Associated Infections and Antimicrobial Resistance (HCAIs & AMR), Department of Infectious Disease, Imperial College, London, UK; 5 Center for Health Policy and Management Studies, Nanjing University, Nanjing, China; 6 The Fourth Affiliated Hospital Zhejiang University School of Medicine, Yiwu, China

## Correction to: Zhen et al. Antimicrob Resist Infect Control (2021) 10:5 https://doi.org/10.1186/s13756-020-00872-w

The original article [1] mistakenly omitted an acknowledgement on behalf of co-author, Nina Zhu. The amended Acknowledgement section ahead displays the full Acknowledgements statement for this article:

### **Acknowledgements**

We want to thank the Center for Health Policy Studies, School of Medicine, Zhejiang University for the assistance in primary data collection. Co-author NZ is funded by the National Institute for Health Research (NIHR) Health Protection Research Unit (HPRU) in Healthcare Associated Infections and Antimicrobial Resistance at Imperial College London in partnership with Public Health England (PHE), in collaboration with Imperial College Health Partners, University of Cambridge and University of Warwick. NZ gratefully acknowledges the support of ESRC as part of the Antimicrobial Cross Council initiative supported by the seven UK research councils, and also the support of the Global Challenges Research Fund. The views expressed in this publication are those of the author(s) and not necessarily those of the NHS, the National Institute for Health Research, the Department of Health and Social Care or Public Health England.
