# Supplementary material for: Economic burden of antibiotic resistance in China: a national level estimate for inpatients
Source: Antimicrob Resist Infect Control. 2021 Jan 6;10:5. doi: 10.1186/s13756-020-00872-w (PMC7789653; doi:10.1186/s13756-020-00872-w)
Supplement: Supplementary file 1 — Additional file 1. [file 13756_2020_872_MOESM1_ESM.docx]

Figure S1. Total population with age and gender distribution in China in 2017[1]

Table S1. Hospital admission rate in China in 2013[2]

| Age (years) | Male (%) | Female (%) |
| --- | --- | --- |
| 65 and over | 20.1 | 19.6 |
| 55-64 | 12.1 | 12.8 |
| 45-54 | 6.8 | 7.7 |
| 35-44 | 4.5 | 6.4 |
| 25-34 | 2.2 | 12.0 |
| 15-24 | 2.0 | 8.0 |
| 5-14 | 2.6 | 1.7 |
| 0-4 | 9.9 | 7.0 |

Table S2. Prevalence of bacterial infection or colonization

| Variables | (%) | Sources |
| --- | --- | --- |
| Total population in China (2017) | Figure S1 | United Nations[1] |
| Hospital admission rate (2013) | Table S1 | China Health Statistical Yearbook[2] |
| Proportion of inpatients with antibiotic prescription (2014) | 38.92 | National Nosocomial Infection Survey[3-5] |
| Proportion of inpatients with bacterial culture detection (2014) | 27.48 | National Nosocomial Infection Survey[3-5] |
| Proportion of inpatients with specimen-confirmed bacterial infection/colonization (2014) | 34.22 | National Nosocomial Infection Survey[3-5] |
| Proportion of single drug resistant infection/colonization among inpatients with positive bacterial culture (2013-2015) | 34.14 | Electronic Medical Records from sampled hospitals |
| Proportion of multiple drug resistant infection/colonization among inpatients with positive bacterial culture (2013-2015) | 46.08 | Electronic Medical Records from sampled hospitals |

Table S3. Standard life expectancy at age of death[1]

| Age | Males | Females |
| --- | --- | --- |
| 0 | 80.00 | 82.50 |
| 1 | 79.36 | 81.84 |
| 5 | 75.38 | 77.95 |
| 10 | 70.40 | 72.99 |
| 15 | 65.41 | 68.02 |
| 20 | 60.44 | 63.08 |
| 25 | 55.47 | 58.17 |
| 30 | 50.51 | 53.27 |
| 35 | 45.57 | 48.38 |
| 40 | 40.64 | 43.53 |
| 45 | 35.77 | 38.72 |
| 50 | 30.99 | 33.99 |
| 55 | 26.32 | 29.37 |
| 60 | 21.81 | 24.83 |
| 65 | 17.50 | 20.44 |
| 70 | 13.58 | 16.20 |
| 75 | 10.17 | 12.28 |
| 80 | 7.45 | 8.90 |
| 85 | 5.24 | 6.22 |
| 90 | 3.54 | 4.25 |
| 95 | 2.31 | 2.89 |

**References**

1. United Nations. World population prospects 2019. 2019. https://population.un.org/wpp/Download/Standard/Mortality/. Accessed 18 August 2019.

2. National Health Commission of the People's Republic of China. 2018 China Health Statistical Yearbook. 2018. http://www.nhc.gov.cn/wjw/tjnj/list.shtml. Accessed April 16 2019.

3. Wu A, Wen X, Li C, Ren N, Gong R, Huang X*, et al***.** China national point prevalence survey on healthcare-associated infection and antimicrobial use in 2012. Chinese Journal of Infection Control**.** 2014; 13:8-15.

4. Wu A, Li C, Wen X, Ren N**.** National healthcare-associated infection surveillance system point-prevalence survey of antimicrobial use in 740 Chinese hospitals in 2010. Chinese journal of Infection Control**.** 2012; 11:7-11.

5. Ren N, Wen X, Wu A**.** Nationwide cross-sectional survey on healthcare-associated infection in 2014. Chinese Journal of Infection Control**.** 2016; 15:83-7.
